# Clinical manifestation, economic burden, and mortality in patients with transthyretin cardiac amyloidosis

**DOI:** 10.1186/s13023-022-02425-3

**Published:** 2022-07-15

**Authors:** Suk-Chan Jang, Jin Hyun Nam, Seung-Ah Lee, Dasom An, Hye-Lin Kim, Sun-Hong Kwon, Eui-Kyung Lee

**Affiliations:** 1 School of Pharmacy, Sungkyunkwan University, 2066 Seobu-ro, Jangan-gu, Suwon, Gyeonggi-do Republic of Korea; 2 Division of Big Data Science, Korea University Sejong Campus, Sejong, Republic of Korea; 3 Division of Cardiology, Department of Internal Medicine, Asan Medical Center, University of Ulsan College of Medicine, Seoul, Republic of Korea; 4 College of Pharmacy, Sahmyook University, Seoul, Republic of Korea

**Keywords:** ATTR-CM, Cardiac amyloidosis, Economic burden, Heart failure, Survival rates

## Abstract

**Background:**

Transthyretin cardiac amyloidosis, also known as transthyretin cardiomyopathy (ATTR-CM) is a poorly-recognized disease with delayed diagnosis and poor prognosis. This nationwide population-based study aimed to identify disease manifestations, economic burden, and mortality of patients with ATTR-CM.

**Methods:**

Data of newly diagnosed patients with ATTR-CM between 2013 and 2018 from the Korean National Health Insurance Service were used, covering the entire population. Patient characteristics included comorbidities, medical procedures, and medication. Healthcare resource utilization and medical costs were observed as measures of the economic burden. The Kaplan–Meier survival curve and years of potential life lost (YPLL) from the general population were estimated for disease burden with ATTR CM.

**Results:**

A total of 175 newly diagnosed patients with ATTR-CM were identified. The most common cardiac manifestation was hypertension (51.3%), while the most common non-cardiac manifestation was musculoskeletal disease (68.0%). Mean medical costs at the post-cohort entry date were significantly higher than those at the pre-cohort entry date ($1,864 vs. $400 per patient per month (PPPM), *p* < 0.001). Of the total medical costs during the study period, the proportion of inpatients cost was 12.9 times higher than the outpatients cost ($1,730 and $134 PPPM, respectively). The median survival time was 3.53 years from the first diagnosis of ATTR-CM, and the mean (SD) YPLL was 13.0 (7.7).

**Conclusions:**

Patients with ATTR-CM had short survival and high medical costs. To reduce the clinical and economic burdens, carefully examining manifestations of disease in patients can help with early diagnosis and treatment.

## Introduction

Transthyretin amyloidosis (ATTR) is caused by the deposition of the misfolded tetrameric protein transthyretin in the peripheral nerves and organs [1]. The signs and symptoms are not specific, and the condition can be insidious as the deposition can occur in any part of the body [2]. The most prevalent areas for deposition are the heart tissues, which cause ATTR-cardiomyopathy (CM), and the peripheral nerves, resulting in ATTR-polyneuropathy (PN) [2]. ATTR-CM is fatal and poorly recognized; it is a late-onset disease with cardiac symptoms generally occurring in patients over 60 years old [1, 3]. The prevalence of ATTR-CM is uncertain, although it is considered rare. In ATTR-CM, TTR amyloid fibrils in the myocardium cause diastolic dysfunction and eventual symptomatic heart failure [4]. A recent study has shown that the number of patients with wild-type ATTR-CM is higher than that of previously diagnosed older people with heart failure [5].

Current treatments for ATTR-CM are limited to symptomatic management through the use of diuretics or pacemakers [1]. Although recently approved disease-modifying drugs such as tafamidis are available, they are not commonly used. Because of the insufficient awareness of ATTR-CM and its non-specific manifestations, delays in its diagnosis are likely, and if left untreated, patients progress to end-stage heart failure with a poor prognosis [6, 7]. Early diagnosis and treatment are critical for a better prognosis before prolonged amyloid deposition, which causes organ dysfunction [6, 7].

A previous study found that diagnosis was delayed for more than 4 years from the onset of cardiac symptoms in 42% of the cases [8]. Another study showed that the hospital inpatient mortality rate was 9.0%, with mean costs of USD 20,584 per patient [9]. Although the diagnosis of ATTR-CM has increased, information on the incidence of ATTR-CM and the demographic characteristics of patients is lacking and may vary by country because of differences in genotype distribution [10]. Additionally, studies examining the medical costs, clinical outcomes, and mortality of the disease are lacking. Therefore, this study aimed to identify the patient manifestations, economic burden, and mortality of patients with ATTR-CM using medical claims data from the Korean National Health Insurance Service (NHIS).

## Methods

### Data source

In this population-based cohort study, we used data from the Republic of Korea NHIS database from 2012 to 2018, which covers the entire national population [11] (approximately 51 million people). The NHIS database was established for health insurance claim reimbursements, and all information on patients’ demographic characteristics, healthcare resource utilization, medical costs, codes for disease diagnosis, date of death, and prescription drugs were included. Disease diagnosis codes were identified according to the International Statistical Classification of Diseases and Related Health Problems 10th Revision (ICD-10, 2016). The data of all patients who were diagnosed with amyloidosis were extracted from the NHIS database for this study.

### Study design and population

**Fig. 1:**
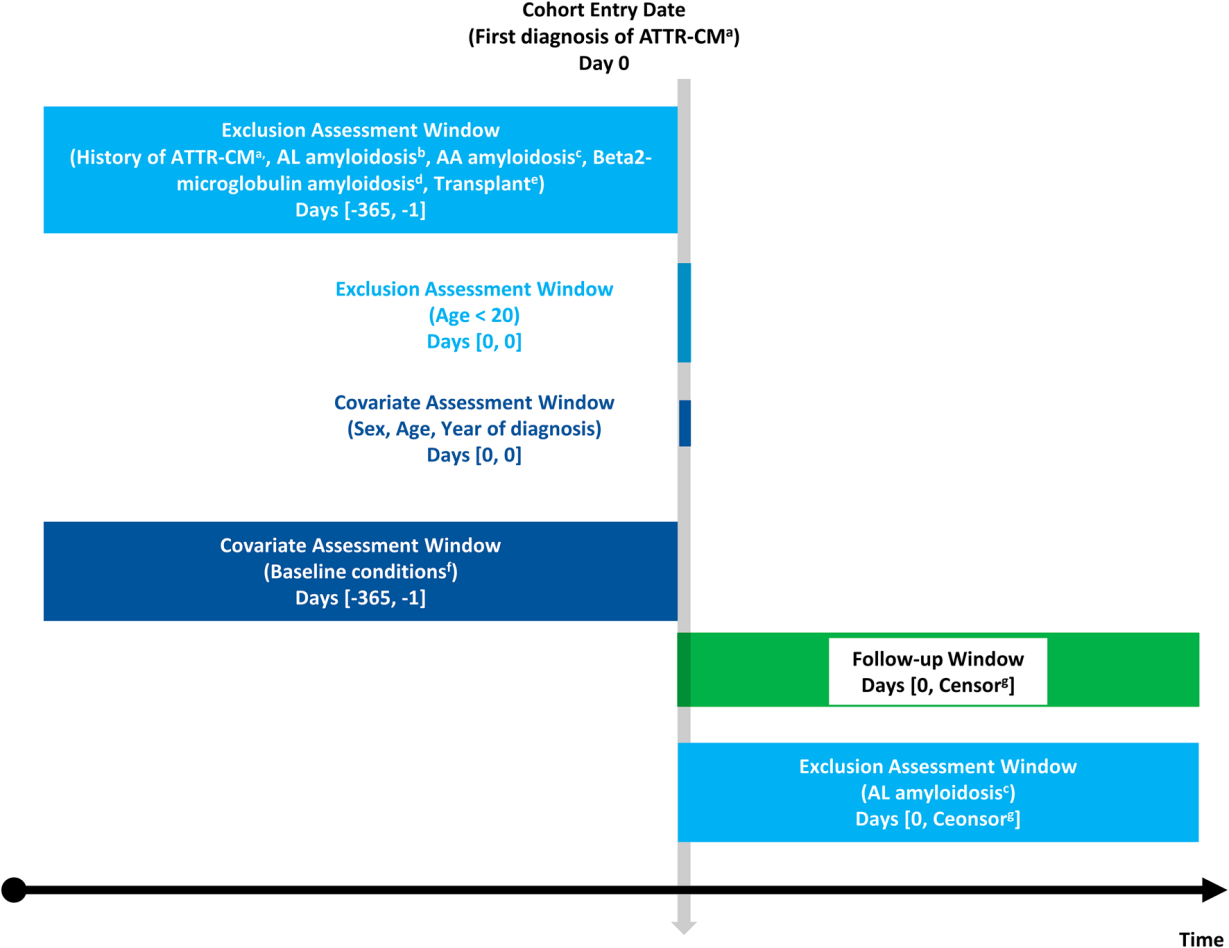
Study design. a Claim from cardiology with ICD-10 code E85; b Chemotherapy, HSCT, multiple myeloma; c connective tissue disease; d hemodialysis; e heart/liver transplant; f CCI, history of diseases; g earliest of: death, lost to follow-up, end of the study. ATTR-CM: Transthyretin amyloid cardiomyopathy, AL: Amyloid light-chain, CCI: Charlson comorbidity index, HSCT: Haematopoietic stem cell transplantation

Patients diagnosed with ATTR-CM were identified by claims from cardiology with amyloidosis (ICD-10 code: E85). The cohort entry window was defined as between January 1, 2013, and December 31, 2018. The cohort entry date was defined as the first day of ATTR-CM diagnosis during the cohort entry window (Fig. 1) [12]. To avoid contamination from prior treatments or any other conditions, the exclusion criteria were defined as follows: 1) patients diagnosed with ATTR-CM within a year before the cohort entry date; 2) amyloid light-chain (AL) amyloidosis (patients diagnosed with multiple myeloma or receiving chemotherapy or hematopoietic stem cell transplantation for the entire study) [13]; 3) AA amyloidosis (patients diagnosed with connective tissue diseases) [13]; 4) Beta2-microglobulin amyloidosis (patients with hemodialysis) [13]; 5) patients who had a heart or liver transplant [14]; and 6) patients under 20 years of age on the cohort entry date. Although several diagnostic tests such as endomyocardial biopsy or nuclear scintigraphy were considered, the claims data for reimbursement included whether diagnostic tests were performed, but not test results. Therefore, we did not include diagnostic tests in the criteria for patients selection. However, the inclusion and exclusion criteria were established and validated referring to the consultation of cardiologists as well as a literature search [9, 15]. The follow-up window was defined as the period from the cohort entry date until the first occurrence of death, loss to follow-up (no claims for more than 6 months), or end of the study.

### ATTR-CM patient characteristics and manifestations

Baseline patient characteristics, including age, sex, year of diagnosis, and type of hospital, were considered for patients undergoing diagnosis from the data recorded on the cohort entry date, and history of the disease was obtained from the data recorded within a year before the cohort entry date. The history of disease included atrial fibrillation (ICD-10 code: I48), hypertension (I10–I13, angiotensin receptor blocker [ARB], or angiotensin-converting enzyme [ACE] inhibitor), other cardiovascular diseases (I20, I21, I25, I63–I70, I73, and I74), musculoskeletal disease (M), depression/anxiety/insomnia (G47 and F except for F00–F03), peripheral neuropathy (G except for G47), diabetes mellitus (E11 and E14), malignancy (C), and dementia (F00–F03), which may have appeared before the ATTR-CM diagnosis. The Charlson comorbidity index (CCI) was calculated as an index of the severity of the underlying disease within a year before the cohort entry date [16].

The procedures and medication for clinical management used during the pre-and post-cohort entry date were also examined. Based on previous studies and clinical practices, we included procedures such as the nerve conduction velocity test, pacemaker, implantable cardioverter defibrillator, tissue or mechanical valve implantation, surgery for spinal stenosis, carpal tunnel syndrome, and heart and liver transplant, along with medication for clinical management including diuretics, mineralocorticoid receptor antagonist (MRA), ARB, statin, ACE inhibitor, digitalis, isosorbide dinitrate, beta-blocker, and hydralazine [5].

### Economic burden of ATTR-CM

To identify disease-related healthcare resource utilization and medical costs across the total burden of patients with ATTR-CM, we compared those that were estimated in the pre- and post-cohort entry dates. The number of inpatient admissions and outpatient visits, the length of hospital stay, and the medical costs were estimated, and the cost values were calculated per patient per month (PPPM). We also calculated the cumulative medical costs considering censoring [17] to show the cost trend after diagnosis. To determine the cost associated with amyloidosis, we categorized the patients into four groups: ATTR-CM-related (claims with amyloidosis and cardiovascular [CV] disease codes), other amyloidosis-related (claims with amyloidosis and without CV disease codes), other CV-related (claims with CV disease and without amyloidosis codes), and non-amyloidosis-related (claims without amyloidosis and CV disease codes). The South Korean Won (KRW) cost was converted to USD (United States Dollar) at the 2018 exchange rate of 1,115.7 KRW/USD.

### Survival rate and years of potential life lost

To assess the mortality and disease burden of patients with ATTR-CM, the survival rate and years of potential life lost (YPLL) were estimated, which are useful measures to recognize the disease burden as they help to quantify premature death [18]. It is important to identify the time from diagnosis to death and determine how quickly the patient will die from the disease. The survival time of patients with ATTR-CM was observed, as well as the number of patients at risk each year. The YPLL in patients with ATTR-CM was also assessed in comparison with the general population. The YPLL is based on the difference between the person’s age at death and the life expectancy of the general population cohort whose age and sex were the same at the time of death. Using the Korean 2019 Complete Life Tables published by the Korean National Statistical Office [19], deceased patients with ATTR-CM were matched with the general population cohort, and the life expectancy was extracted.

### Statistical analysis

To calculate the survival rate, survival analysis was performed to estimate the survival probability over time. The median survival time of patients with ATTR-CM and the number of patients at risk each year were calculated using a Kaplan–Meier curve with a 95% confidence interval. The age at diagnosis, age at death, and YPLL were stratified according to age group, sex, CCI, and history of diseases. All YPLLs, healthcare utilization, and medical costs were calculated as means and standard deviations (SDs). We conducted a paired t-test to compare the differences between healthcare utilization and medical costs before and after diagnosis. A weighted available sample estimator was applied when calculating the cumulative medical cost because censoring is a frequent limitation when working with event data, especially in cost analysis [17]. Using the weighted available sample estimator, the mean costs were calculated by correcting the censored data. All statistical analyses were performed using R (version 4.1; The R Foundation for Statistical Computing, Vienna, Austria) and SAS (version 9.4; SAS Institute, Cary, NC, USA).

## Results

### Patient characteristics

**Fig. 2:**
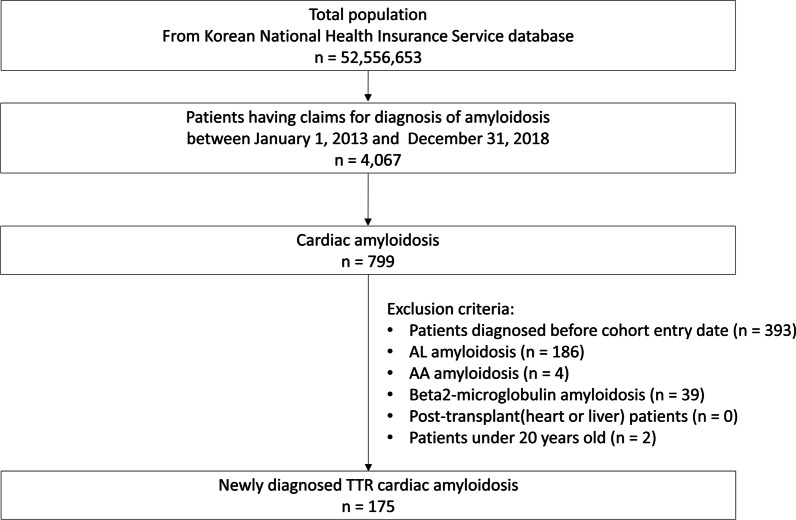
Flow chart of the selection process for eligible patients with ATTR-CM. ATTR-CM: Transthyretin amyloid cardiomyopathy, AL: Amyloid light-chain

A total of 4,067 people were diagnosed with amyloidosis between 2013 and 2018, and 799 patients had cardiac amyloidosis (Fig. 2). A total of 175 newly diagnosed patients with ATTR-CM were included in the analysis. The mean follow-up window of the study was 1.5 years. The baseline characteristics of the patients included in this study are shown in Table 1. The mean age of the population was 69.3 years, and there were more males (n = 110, 62.9%) than females. Most patients were diagnosed with ATTR-CM in tertiary hospitals (82.3%). The rate of diagnosis continued to increase from 14 patients (8.0%) in 2013 to 46 patients (26.3%) in 2018. In addition to heart failure, 51.3% of patients had hypertension, 20.6% had atrial fibrillation, and 54.3% had other cardiovascular diseases. Musculoskeletal disease (68.0%) was the most common non-cardiac manifestation, followed by depression/anxiety/insomnia (44.0%) and peripheral neuropathy (33.7%).

**Table 1:** Baseline characteristics of patients newly diagnosed with ATTR-CM

Patients newly diagnosed with ATTR-CM (n = 175)	
Age (years), mean (SD)	69.3(13.5)
< 65, n (%)	48 (27.4)
≥ 65	127 (72.6)
Sex, n (%)	
Male	110 (62.9)
Female	65 (37.1)
Year of diagnosis, n (%)	
2013	14 (8.0)
2014	29 (16.6)
2015	21 (12.0)
2016	28 (16.0)
2017	37 (21.1)
2018	46 (26.3)
Type of hospital undergoing diagnosis, n (%)	
Tertiary hospital	144 (82.3)
General hospital	31 (17.7)
CCI, mean (SD)	2.71 (2.01)
< 3	87 (49.7)
≥ 3	88 (50.3)
History of diseases, n (%)	
Cardiac manifestation	
Hypertension	90 (51.3)
Atrial fibrillation	36 (20.6)
Other cardiovascular diseases	95 (54.3)
Non-cardiac manifestation	
Musculoskeletal disease	119 (68.0)
Depression, anxiety, insomnia	77 (44.0)
Peripheral neuropathy	59 (33.7)
Diabetes mellitus	57 (32.6)
Malignancy	17 (9.7)
Dementia	9 (5.1)

*ATTR-CM* Transthyretin amyloid cardiomyopathy, *CCI* Charlson comorbidity index, *SD* Standard deviation

The nerve conduction velocity test showed the highest number of procedures both before and after diagnosis, and a high rate of increase after diagnosis (6.3% and 17.1%, respectively) (Table 2). Pacemakers and implantable cardioverter-defibrillator were performed the next most after diagnosis (4.0% and 2.3%, respectively). Carpal tunnel syndrome surgery was performed more before diagnosis than after diagnosis (2.9% and 1.1%, respectively). The most commonly used medications were diuretics (85.1%), followed by MRA (53.7%), ARB (45.7%), statins (35.4%), and ACE inhibitors (21.7%).

**Table 2:** Procedures and medication for clinical management of patients with ATTR-CM

	Pre-cohort entry date (n = 175)	Post-cohort entry date (n = 175)
*Procedures, n (%)*		
Nerve conduction velocity test	11 (6.3)	30 (17.1)
Pacemaker	2 (1.1)	7 (4.0)
Implantable cardioverter defibrillator	-	4 (2.3)
Surgery for spinal stenosis	3 (1.7)	2 (1.1)
Surgery for carpal tunnel syndrome	5 (2.9)	2 (1.1)
Liver transplant	–	1 (0.6)
Heart transplant	–	1 (0.6)
Tissue or mechanical valve implantation	–	–
*Medication for clinical managements, n (%)*		
Diuretic	111 (63.4)	149 (85.1)
MRA	67 (38.3)	94 (53.7)
ARB	73 (41.7)	80 (45.7)
Statin	57 (32.6)	62 (35.4)
ACE inhibitor	29 (16.6)	38 (21.7)
Digitalis	25 (14.3)	28 (16.0)
Isosorbide dinitrate	12 (6.9)	20 (11.4)
Beta-blocker	17 (9.7)	12 (6.9)
Hydralazine	–	1 (0.6)

*ACE* Angiotensin-converting enzyme, *ARB* Angiotensin receptor blocker, *ATTR-CM* Transthyretin amyloid cardiomyopathy, *MRA* Mineralocorticoid receptor antagonist, *SD* Standard deviation

### Economic burden of ATTR-CM

**Fig. 3:**
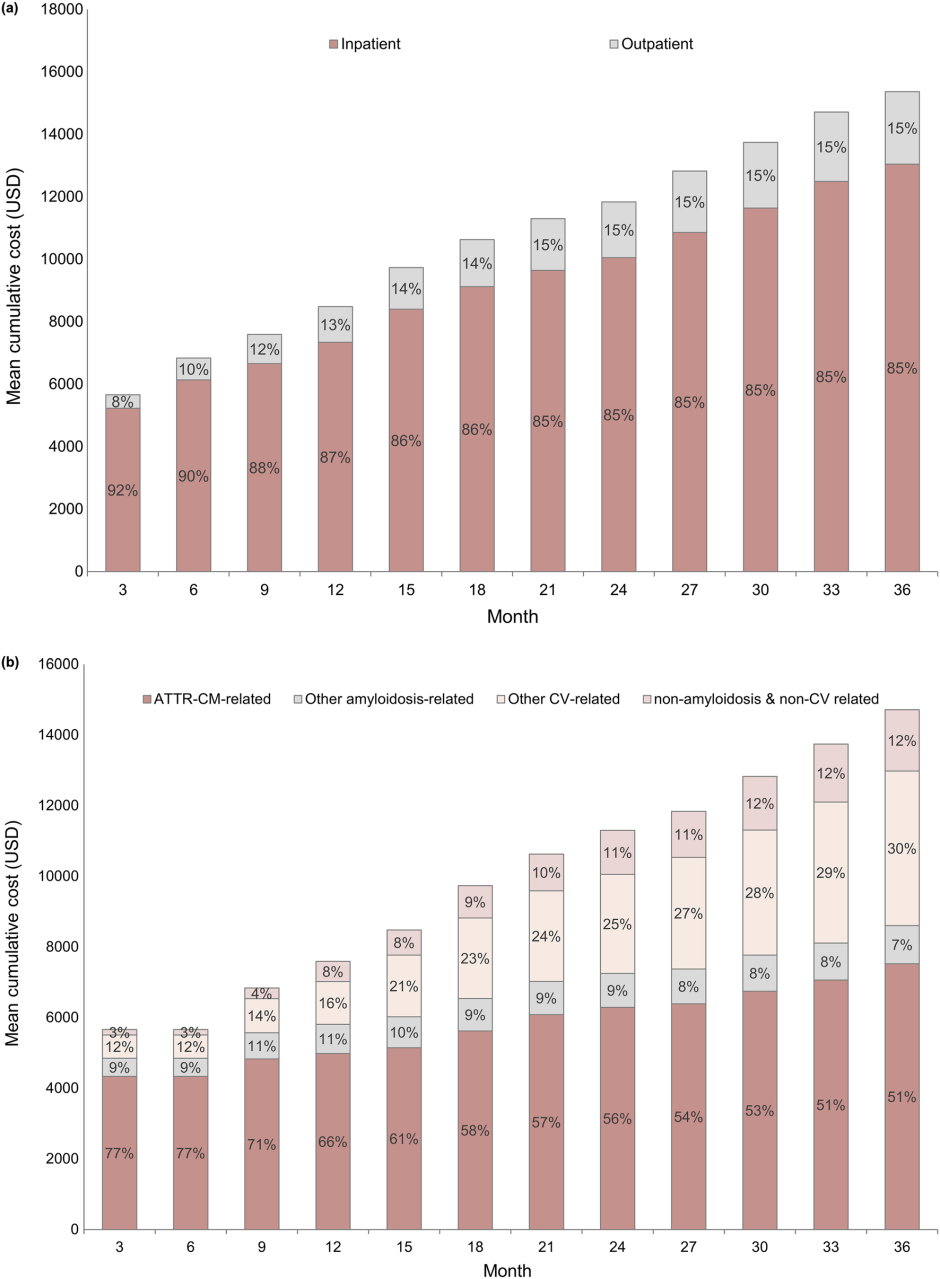
Cumulative medical costs of patients with ATTR-CM. **a** Inpatient and outpatient costs; **b** disease-related costs. ATTR-CM: Transthyretin amyloid cardiomyopathy, CV: Cardiovascular

Table 3 shows the healthcare utilization and medical costs for patients with ATTR-CM during the pre- and post-cohort entry dates. The total number of inpatient admissions was 3.8 times higher in the post-cohort entry date than in the pre-cohort entry date (mean [SD], 0.38 [0.55] vs. 0.10 [0.12] PPPM, *p* < 0.001). In addition, the day length of hospital stay (in days) was 5.1 times longer in the post-cohort entry date than in the pre-cohort entry date (5.72 [9.08] vs. 1.12 [2.78], *p* < 0.001). The total follow-up cost was $1,864 PPPM ($1,730 and $134, inpatient and outpatient, respectively) in the post-cohort entry date, and $400 PPPM ($313 and $87, respectively) in the pre-cohort entry date. The costs for the pre- and post-cohort entry dates showed statistically significant differences (*p* < 0.001). Figure 3 shows the cumulative medical costs corrected for the censored data. Inpatient costs accounted for the majority of the total medical costs, but their proportion decreased until 1.5 years after diagnosis (Fig. 3a). The ATTR-CM-related cumulative cost was the highest, followed by other CV-related costs (Fig. 3b). The ratio of ATTR-CM-related costs continued to decrease, while CV-related costs showed a tendency to increase. The other amyloidosis-related cumulative costs remained constant.

**Table 3:** Healthcare utilization and medical costs of patients with ATTR-CM (PPPM)

	Pre-cohort entry date, mean (SD)	Post-cohort entry date, mean (SD)	*P*-value
Number of inpatient admissions	0.10 (0.12)	0.38 (0.55)	< 0.001
ER	0.01 (0.03)	0.03 (0.11)	0.040
ICU	0.01 (0.04)	0.09 (0.27)	< 0.001
Length of hospital stay (days)	1.12 (2.78)	5.72 (9.08)	< 0.001
Number of outpatient visits	2.63 (2.10)	2.60 (1.96)	0.837
ER	0.02 (0.06)	0.05 (0.25)	0.085
Follow-up costs	$400 ($588)	$1864 ($2773)	< 0.001
Inpatient	$313 ($567)	$1730 ($2767)	< 0.001
Outpatient	$87 ($76)	$134 ($154)	< 0.001

*ATTR-CM* Transthyretin amyloid cardiomyopathy, *ER* Emergency room, *ICU* Intensive care unit, *PPPM* Per patient per month, *SD* Standard deviation

### Survival rates and YPLL

**Fig. 4:**
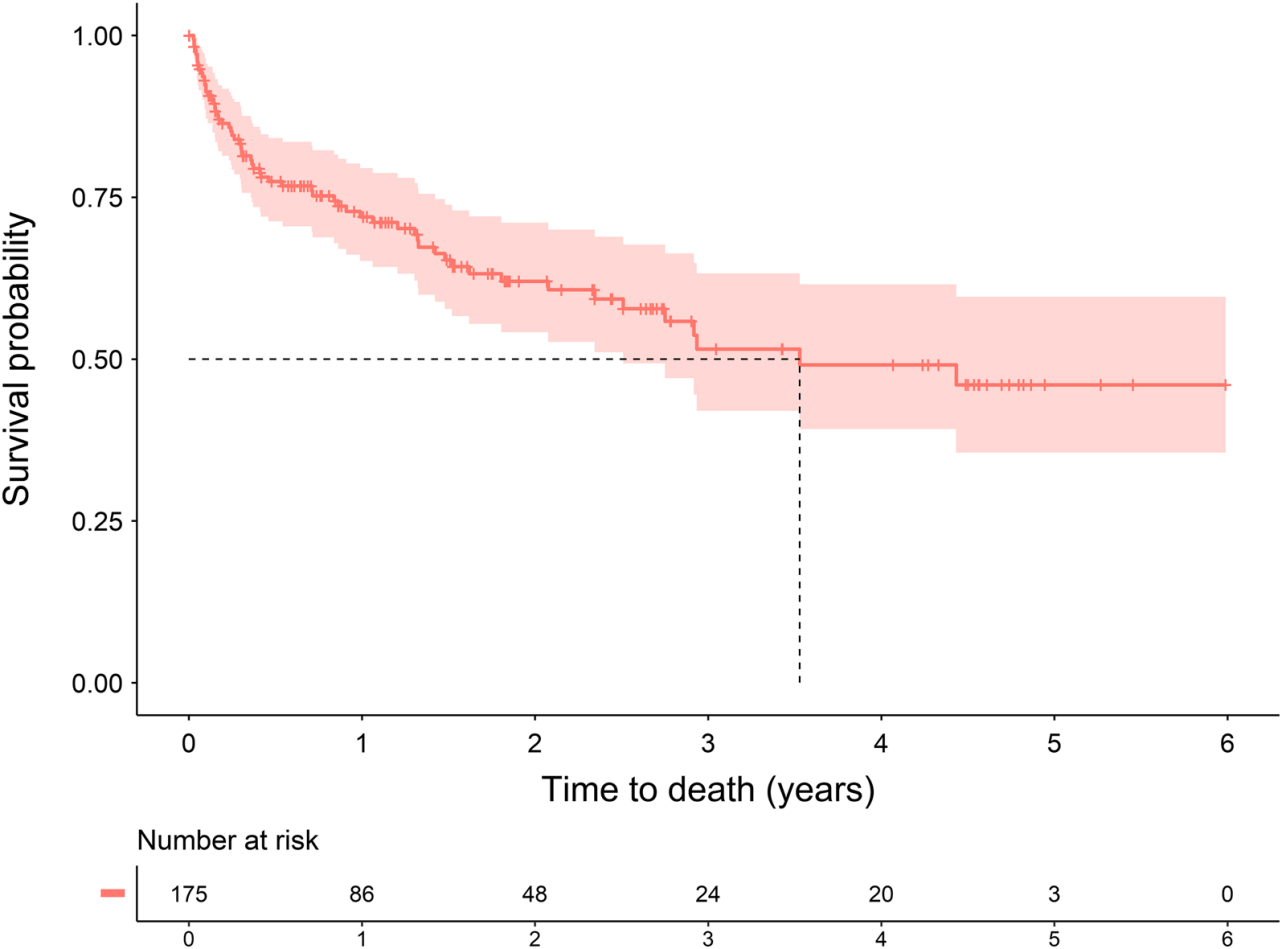
Survival probability of patients with ATTR-CM. ATTR-CM: Transthyretin amyloid cardiomyopathy

The median survival time of patients with ATTR-CM was 3.53 years (Fig. 4). Among 175 patients, 62 patients died, and the mean YPLL was 13.0 years (Table 4). The group of patients aged under 65 years had a higher YPLL (27.4 years) than those aged 65 years or older (10.5 years). Women had 14.6 YPLL, whereas men had 12.0 YPLL. Regarding the history of diseases, 13 of 36 patients with atrial fibrillation died, resulting in 10.6 YPLL; 33 of 90 patients with hypertension died, resulting in 11.5 YPLL; and 36 of 95 patients with other cardiovascular diseases died, resulting in 13.5 YPLL. In the case of peripheral neuropathy, 29 of 59 patients died, and their YPLL was 12.8.

**Table 4:** Years of potential life lost (YPLL) stratified by selected characteristics among patients with ATTR-CM compared to an age and sex-matched general population (years)

	No. of patients	No. of death, n (%)	Age at diagnosis, mean (SD)	Age at death, mean (SD)	YPLL (years), mean (SD)
Total	175	62 (35.4)	75.2 (10.6)	75.9 (10.5)	13.0 (7.7)
Age group (years)					
< 65	48	9 (18.8)	56.8 (3.8)	57.8 (4.3)	27.4 (5.0)
≥ 65	127	53 (41.7)	78.3 (7.8)	79.0 (7.7)	10.5 (4.8)
Sex					
Male	110	38 (34.5)	75.1 (10.9)	76.0 (10.7)	12.0 (7.0)
Female	65	24 (36.9)	75.3 (10.3)	75.9 (10.3)	14.6 (8.6)
CCI					
< 3	87	21 (24.1)	69.9 (11.3)	71.1 (11.2)	16.4 (9.2)
≥ 3	88	41 (46.6)	77.9 (9.2)	78.4 (9.2)	11.2 (6.2)
History of diseases					
Cardiac manifestation					
Hypertension	90	33 (36.7)	77.2 (8.3)	77.9 (8.4)	11.5 (5.9)
Atrial fibrillation	36	13 (36.1)	78.3 (8.6)	79.1 (8.2)	10.6 (4.5)
Other cardiovascular disease	95	36 (37.9)	75.3 (11.2)	76.1 (11.1)	13.5 (8.4)
Non-cardiac manifestation					
Musculoskeletal disease	119	52 (43.7)	76.3 (10.4)	77.1 (10.2)	12.3 (7.3)
Depression, anxiety, insomnia	77	34 (44.2)	75.9 (9.9)	76.6 (9.8)	12.8 (7.4)
Peripheral neuropathy	59	29 (49.2)	75.7 (8.8)	76.4 (8.5)	12.8 (6.5)
Diabetes mellitus	57	22 (38.6)	75.7 (9.1)	76.5 (9.3)	12.7 (7.3)
Malignancy	17	5 (29.4)	83.4 (3.1)	84.4 (2.5)	7.4 (1.8)
Dementia	9	9 (100.0)	83.6 (7.7)	83.9 (7.6)	7.9 (3.7)

*ATTR-CM* Transthyretin amyloid cardiomyopathy, *CCI* Charlson comorbidity index, *SD* Standard deviation, *YPLL* Years of potential life lost

## Discussion

In this retrospective population-based study, the survival rates, YPLL, and clinical and economic burdens were estimated for patients with ATTR-CM. We used NHIS data, consisting of almost the entire national population, as a population-based representative data source. We found that the economic burden of patients with ATTR-CM, especially inpatient costs, was approximately ten times higher than that of other heart failures [20, 21]. The survival rates and YPLL in long-term real-world data were assessed and compared to previous studies. The median survival time was 3.53 years, and the mean YPLL was 13.0 years.

This study showed the clinical and economic burdens of the ATTR-CM. Compared to before diagnosis, inpatient costs were much higher than outpatient costs, a trend that was noticeable immediately after diagnosis. This high cost appeared probably because the prognosis was poor at the time of diagnosis due to delays in diagnosis and misdiagnoses [7, 22]. A previous study that analyzed hereditary TTR amyloidosis showed a similar result, with very high inpatient cost compared to outpatient cost, although the slope of the trend in our study was slightly different [15]. This is presumed to be because the previous study contained several patients with ATTR-PN, and ATTR-PN has a relatively good prognosis compared to ATTR-CM. Other amyloidosis-related costs are incurred only immediately after diagnosis and are rarely incurred afterward, which is estimated to be the cost of other amyloidoses such as PN-related diagnostic tests other than CM. We also found much higher medical costs than other Korean studies on heart failure, suggesting that ATTR-CM has symptoms similar to heart failure, but is much more difficult to manage [20, 21].

Patients with ATTR-CM require management of heart failure symptoms, but ARBs, ACE inhibitors, and beta-blockers are not widely used in ATTR-CM compared to other heart failures because patients with ATTR-CM do not tolerate these drugs well due to the presence of hypotension [5]. Instead, well-tolerated loop diuretics and MRA were used in most patients. We also found that many patients had non-cardiac manifestations as well as cardiac manifestations at the time of diagnosis. As effective ATTR-CM therapies gradually emerge and are introduced, it is a need to optimize treatment by carefully monitoring disease manifestations to increase suspicion of the disease and diagnosis early [5]. Although such therapies have not yet been introduced in Korea, it is expected that the prognosis of patients will be improved if new disease-modifying drugs are introduced.

The diagnostic delay of ATTR-CM due to under-recognition is a continuing clinical challenge [23, 24]. This study captured the actual clinical practice of under-recognized disease by using population-based real-world data. The median survival time of 3.53 years found in this study was shorter than that found by Lane et al*.* [8] (4.75 years for wild-type ATTR-CM, 2.58 years for V122I-hereditary ATTR-CM, and 5.75 years for non-V122I-hereditary ATTR-CM). The prognosis of V122I-hereditary ATTR-CM was worse than that in our study, but considering the genotype rate identified in previous studies, it is predicted that the rate of the V122I mutation included in our study may be very low [10, 25]. In particular, we expect that there were many cases of diagnostic delay in Korea due to the lack of medication, which has been indicated by cardiologists to be similar in actual clinical practice. Because our study used nationwide data covering the entire population, it better reflects the true overall diagnosis timelines than a study that analyzed only a small number of patients at specific centers.

Patients with ATTR-CM died approximately 13.0 years earlier than the general population. In particular, the YPLL was high in the group aged < 65 years; this is presumed to be because hereditary ATTR-CM is usually diagnosed in the young and has a poorer prognosis [26, 27]. In addition, even if the disease onset was early, the time to death was similar to that of the elderly group, and therefore, the YPLL was high. There was no significant difference in survival between men and women, and although women had a longer life expectancy, female patients had higher YPLL than male patients [8]. Although the YPLL differed according to the history of diseases, considering the age at diagnosis, it was due to age.

The average age at diagnosis was approximately 70 years, and the predominance of males was similar to that in previous studies [5, 8]. ATTR-CM is still an underdiagnosed condition, but as recognization increases, the number of diagnoses is also steadily increasing. Many patients had cardiovascular diseases in addition to heart failure, but fewer had hypertension compared to the general population [28]. It was presumed that the natural cure of hypertension was a typical sign of ATTR-CM [5]. Carpal tunnel syndrome is a well-known implication of ATTR-CM [29]. Many patients had a history of musculoskeletal diseases, including carpal tunnel syndrome. One-third of patients had peripheral neuropathy, a common prognostic symptom of ATTR-CM [5].

Despite the significance of the results, this study has some limitations. As there is no clear ICD-10 code representing ATTR-CM, the operational definition was used to find ATTR-CM cases, and therefore, some other amyloidoses could be present in our analysis. To compensate for this, we tried to select ATTR-CM exclusively by broadly including cardiac amyloidosis and excluding patients with other cardiac amyloidoses such as AL amyloidosis, AA amyloidosis, and Beta-2-microglobulin amyloidosis. Since ATTR-CM is an orphan disease and is not well-known, we validated various possible criteria through literature review and consultations with cardiologists. Another limitation relates to the use of claims data in the absence of clinical information, including genotype, clinical red flags, and diagnostic test results such as biopsy and nuclear scintigraphy. Moreover, the genotype of hereditary ATTR-CM could affect survival and show different outcomes compared to other countries’ studies because the genotypic ratio varies from country to country [3, 10]. Clinical red flags for ATTR-CM, such as left ventricular thickness, QRS voltage, echocardiographic hypertrophic phenotype, and troponin levels, could not be included, although these factors might have an impact on the ATTR-CM diagnosis [30].

## Conclusion

A short survival rate and high medical cost in patients with ATTR-CM were reaffirmed in comparison with previous studies. This high clinical and economic burden of patients with ATTR-CM is likely caused by diagnostic delays in real-world clinical practice. Carefully examining patient manifestations can help with early diagnosis and treatment. As new disease-modifying treatments have been approved recently, early diagnosis and treatment may improve prognosis and alleviate the economic burden in patients with ATTR-CM.

## Data Availability

Data from the Korean National Health Insurance Service were used and were obtained after appropriate authorisation approval.

## Abbreviations

ACE: Angiotensin-converting enzyme
AL: Amyloid light-chain
ARB: Angiotensin receptor blocker
ATTR: Transthyretin amyloidosis
CCI: Charlson comorbidity index
CM: Cardiomyopathy
CV: Cardiovascular
MRA: Mineralocorticoid receptor antagonist
NHIS: National Health Insurance Service
PN: Polyneuropathy
PPPM: Per patient per month
YPLL: Years of potential life lost
